# Clinical and radiographic evaluation of NeoMTA versus conventional white mineral trioxide aggregate in revascularization of non-vital immature permanent anterior teeth (A randomized controlled trial)

**DOI:** 10.1038/s41405-023-00143-x

**Published:** 2023-04-28

**Authors:** Hala Ahmed Tawfeek, Adel Abdel-Azim El-Bardissy, Mohammed Abou El-Yazeed, Randa Youssef, Ahmed Mohamed Abd Alsamad

**Affiliations:** 1 Pediatric Dentistry, Orthodontics and Pediatric Dentistry Department, Oral and Dental Research Institute, National Research Centre, Giza, Egypt and Phd student in Pediatric Dentistry and Dental Public Health, Faculty of Dentistry, Cairo University, Giza, Egypt; 2 Pediatric Dentistry and Dental Public Health, Faculty of Dentistry, Cairo University, Giza, Egypt; 3 Pediatric Dentistry, Orthodontics and Pediatric Dentistry Department, Oral and Dental Research Institute, National Research Centre, Giza, Egypt; 4 Oral and Maxillofacial Radiology, Faculty of Dentistry, Cairo University, Giza, Egypt

**Keywords:** Paediatric dentistry, Mineral trioxide aggregate

## Abstract

**Objective:**

To evaluate and compare clinically and radiographically the effect of using two different coronal plug materials (NeoMTA versus Conventional White mineral trioxide aggregate) in revascularization of non-vital immature permanent anterior teeth, with special reference to the assessment and evaluation of discoloration potential over a period of one year.

**Methods:**

Revascularization procedure was performed in (30) immature permanent non-vital anterior teeth which were randomly allocated to two equal groups (*n* = 15). NeoMTA was used as coronal plug material in the Experimental Group (N), while conventional White mineral trioxide aggregate (WMTA) was used as a coronal plug material in the Control Group (W). All treated teeth were evaluated clinically at 1 week, 1, 3, and 12 months and radiographically at 12 months.

**Results:**

The overall clinical and radiographic success rate of Groups (N) and (W) at the end of the 12-month follow-up period was 100%. The discoloration was detected in a single tooth (9.1%) in Group (N) and three teeth (27.3%) in Group (W) but the difference between groups was not statistically significant.

**Conclusions:**

Both NeoMTA and conventional WMTA were successful coronal plug materials in the revascularization of non-vital immature permanent teeth achieving a high level of clinical and radiographic success. NeoMTA is a promising coronal plug material that can be used for revascularization procedures in the esthetic zone as it showed less discoloration potential compared with conventional WMTA, however, there was no statistically significant difference between both materials.

## Introduction

Pulp necrosis of immature permanent teeth is considered a critical condition because it leads to the arrest of root development and incomplete dentin deposition leaving a weak fragile root that is more liable to fracture, it will also lead to a poor crown/root ratio with a possible periodontal breakdown [1–3].

Several techniques have been used for the management of non-vital immature permanent teeth including calcium hydroxide apexification and apical plug technique. Although these techniques were successful in achieving apical closure and healing of the periapical pathosis, they do not contribute to any quantitative or qualitative increase in root dimensions since a hard tissue barrier formation only occurs apically without further root development [2].

Revascularization is a regenerative endodontic procedure (REP) that stimulates the continuation of root development. It is considered a valuable treatment as it strengthens the root walls by stimulating the deposition of hard tissues and promoting the development of normal apical morphology [4, 5].

Revascularization treatment is based on the postulation that is creating an environment free of microorganisms with a proper bacterial-tight seal in the presence of an appropriate three-dimensional scaffold, stem/progenitor cells inside the root canal; stimulates tissue repair in nonvital immature permanent teeth [1, 3, 6].

Mineral trioxide aggregate (MTA) has been widely used in revascularization procedures for coronal sealing in more than 85% of studies because of its biocompatibility, good sealing properties, and marginal adaptation. However, its poor handling characteristics and potential coronal discoloration effects are the major disadvantages of using white mineral trioxide aggregate (WMTA) [7, 8].

The NeoMTA (NuSmile, Huston, USA) is pure MTA that is marketed as a cost-effective MTA intended to be used for pediatric pulp therapy as it has fast setting time, easy handling and most important modification is its non-staining formulation. Tantalum oxide (Ta_2_O_5_) has been added to NeoMTA as a radiopacifying agent instead of bismuth oxide (Bi_2_O_3_) which has been linked mainly to the cause of discoloration in conventional mineral trioxide aggregate [8, 9].

Revascularization of immature permanent teeth has become an important part of endodontic treatment modalities. Despite its high success rate clinically and radiographically, many studies have reported that discoloration is a significant esthetic concern following revascularization procedure as appearance and pleasing esthetics are patient-centered outcomes [7].

Most currently available clinical trials mainly focus on clinical and radiographic outcomes of materials in addition to reporting their discoloration potential only in binary ways. Available data concerning the quantitative assessment of discoloration of new materials are from in-vitro studies bear in mind the difference in a clinical scenario than under controlled in-vitro conditions. So, this study aimed to evaluate clinically and radiographically the effect of using two types of coronal plug materials in the revascularization of non-vital immature permanent anterior teeth with special reference to assessment and evaluation of discoloration potential over a period of one year.

## Subjects and methods

The Research Ethics Committee, Faculty of Dentistry, Cairo University approved the protocol for this parallel, double-blinded, randomized controlled trial with a 1:1 allocation ratio with (reference no.18/7/52). The study has been registered on Clinicaltrials.gov with identification number: NCT03545139, under the title Clinical & Radiographic Evaluation of NeoMTA Versus Conventional White MTA in Revascularization of Non-Vital Immature Permanent Teeth. The trial was conducted from September 2019 to January 2021.

### Sample size determination

Sample size determination was done before the study using R statistical package version 3.3.1. The T-test power calculation was used to detect the proper sample size based on the results of Lenherr et al. [10]. A total sample size of 22 (teeth) was found to be adequate to detect a mean difference in color change between study groups of 3.5 points (SD = ± 2.44) with a power of 90% and a two-sided significance level of 5%; with equal allocation to two arms (11 teeth in each group). To compensate for 30% non-response rate or possible patient attrition, the sample was increased to a total size of 30 (teeth) that were divided into 2 groups.

### Study setting

- A total of 30 non-vital immature permanent anterior teeth in 25 children were enrolled in this study from the outpatient clinic of the Pediatric Dentistry Department, Faculty of Dentistry, Cairo University.
- The procedures were carried out by the main investigator for all patients.

### Eligibility criteria

#### Inclusion criteria

- 8–15 years old children.
- Free from any systemic diseases.
- Upper traumatized permanent anterior teeth with non-vital pulp and immature root apex.
- Pulp space not needed for post and core.

#### Exclusion criteria

- Poor oral hygiene.
- Teeth with root resorption, luxation injuries, and root fracture.
- Teeth with severe discoloration/ unacceptable color difference between affected tooth and contralateral tooth (ΔE ≥ 5).

### Pre-operative protocol

**Fig. 1:**
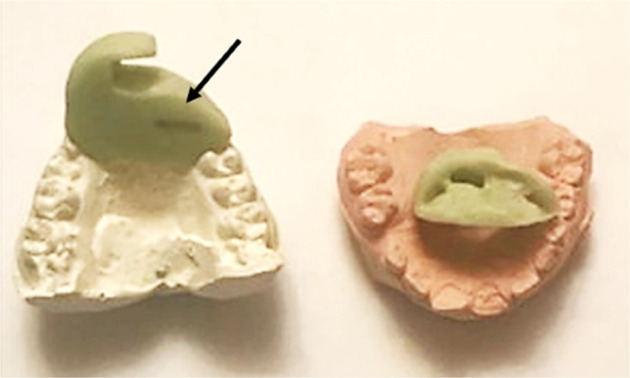
Radiographic stent (acrylic bite block) and shadow of the embedded stainless-steel wire appearing.

**Fig. 2:**
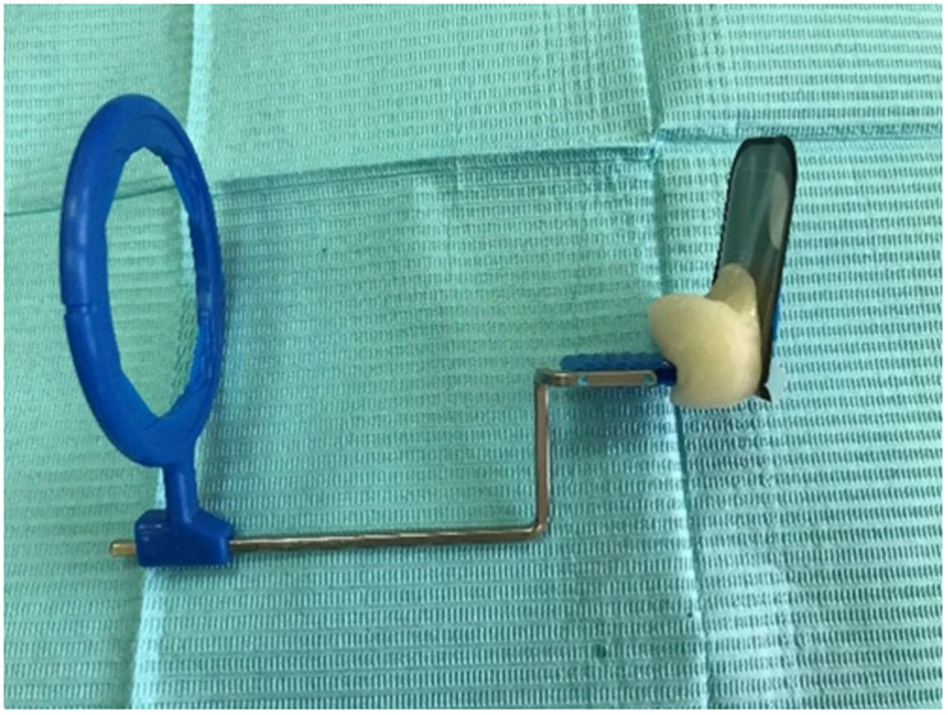
Individualized extension cone paralleling index.

- Personal, medical, dental, trauma history, and clinical examination were attained based on the checklist of the European Society of Endodontology for revitalization’s pre-operative diagnostic procedures [1].
- Conventional pre-operative periapical radiograph was taken.
- Detailed procedures, benefits, and expected harms were discussed with the child’s legal guardian, then informed consent was obtained.
- Preparation for digital radiographic procedures and construction of radiographic stent was done to perform an individualized Extension Cone Paralleling (XCP) index for each patient according to Aly et al. [2]. A radio-opaque object of known dimension (5 mm stainless steel wire) was embedded in the acrylic stent before setting [11] as shown in Figs. 1, 2.

### Randomization and allocation concealment

The 30 permanent immature teeth were randomly assigned by simple randomization procedure into two equal groups, Experimental Group (N): 15 teeth treated with NeoMTA (NuSmile Neo MTA ^TM^) and Control Group (W): 15 teeth treated with Conventional WMTA (White Angelus MTA) using shuffled closed white opaque envelopes picked by a patient at the second appointment just before placement of the coronal plug step.

### Blinding

- The participants and legal guardians were blinded.
- Radiographic assessor, one of the clinical assessors for the color change to avoid detection bias, and the statistician were blinded to avoid reporting bias.

### Intraoperative procedures

Treatment of the selected teeth was performed according to AAE [12] clinical considerations for a regenerative procedure, same procedures were applied to all teeth in the study the only difference was the coronal plug material used.

#### First appointment for revascularization procedure

Each tooth was locally anesthetized using topical anesthesia gel benzocaine 20% followed by labial infiltration using Articaine HCL 4% with 1:100,000 epinephrine then isolated with a rubber dam. A conventional access cavity was done then the working length was determined radiographically. Passive pressure irrigation using 1.5% sodium hypochlorite (NaOCl) (20 mL/canal, 5 min) with a side vent needle placed 1–2 mm from the apex, then sterile physiological saline (5 mL), followed by 17% Ethylenediaminetetraacetic acid (EDTA) (20 mL/canal, 5 min). Canal dryness was performed using suitable size paper points then placement of intracanal medication (double antibiotic paste) prepared from equal amounts of Ciprofloxacin 500 mg and Metronidazole 500 mg with a ratio of 1:1. The mix was delivered into the canal using a disposable plastic syringe having plastic tips adjusted to be 2 mm shorter than the working length. The excess paste was removed, and the access cavity was sealed with dry cotton and 3–4 mm of light-cured resin-modified glass ionomer (RMGI) (Riva, SDI, Australia) then the patient was dismissed for 4 weeks.

#### Second appointment: (4 weeks after the First appointment)

After 4 weeks, the response to the initial treatment done at the first appointment was assessed.

##### Criteria of clinical success or failure of the first appointment according to the AAE [12]

Complete resolution of signs and symptoms which include pain, swelling, sinus, or fistula was considered success of the first appointment.

##### Tooth color assessment

1. Tooth color baseline T_0_ was recorded for the affected tooth at the beginning of the second appointment using VITA Easyshade V digital spectrophotometer, three measurements were recorded then the mean color was calculated.
2. Intraoral photographs were captured for documentation using fixed settings of the camera with no flash [13].

##### Operating procedures

Each tooth was locally anesthetized using topical gel and labial infiltration using 3% mepivacaine without vasoconstrictor. The temporary dressing was removed after isolation by rubber dam then irrigation with 17% (EDTA) (20 mL/canal, 5 min) followed by canal dryness. Bleeding was obtained by over-instrumenting and rotating a pre-curved K-file at 2 mm past the canal to have the entire canal filled with blood. A tight cotton pellet slightly wetted with sterile saline was inserted and left for 10–15 min to allow the formation of a clot then cleaning of any blood remnants on the walls of the cavity was done using a bond brush. A resorbable collagen matrix Colla-plug™ (Zimmer Dental Inc. Aston Avenue Carlsbad, CA, USA) was placed over the clot followed by placement of the coronal plug material according to the tooth allocated in which group, either NeoMTA (NuSmile Neo MTA™.) or conventional WMTA (White Angelus MTA. Angelus Indústria de Produtos Odontológicos. Waldir Landgraf Street- Londrina- Brazil) forming about 3 mm thickness just underneath the cementoenamel junction (CEJ). Excess material on the cavity wall was removed, then a conventional periapical radiograph was taken to double-check the proper position of the coronal plug in relation to CEJ. Once the material became firm within 10–15 min, RMGI followed by composite filling (3 M, Filtek, Z350, United Kingdom) was placed. Immediate postoperative (baseline) digital radiograph was taken at the end of the second appointment using a Digital x-ray machine (Minray, Soredex, Tuusula); using a standardized paralleling technique by the (XCP) alignment system with the radiographic stent and the large 3 × 4 cm phosphor storage plates (PSPs) imaging plate (Soredex DIGORA®, Finland). The DIGORA Optime scanner scanned imaging plates.

- All patients were planned to be recalled for clinical follow-up after 1 week, 1 month, 3 months, 6 months, and 12 months while radiographic follow-up was planned to be at 6 months and 12 months as shown in Table 1, but due to the Covid-19 lockdown, a modified follow up was done as all patients were not able to attend the six months recall visit. Consequently, the following measures were done to assure the patients and their parents. Through a phone call, parents were asked about pain, and change in color if present. Parents were taught how to examine visually the vestibule. Moreover, parents were requested to send an intra-oral photo if possible. All the data that were collected during this period were just to check on patients but were not used for statistical analysis.

**Table 1:** Participant timeline for enrollment, treatment procedures and follow up.

		Study period								
		Enrolment	Treatment procedures			Follow up				Close out
Time point			_1st appointment_	_2nd app 4 w_ (T_0_)	_4w_ (Resistant cases)	_T1 1w_	_T2 1m_	T_3 3m_	_T4 6m_	T_x 12m_
Enrolment	Eligibility screen	X								
	Verbal interview	X								
	Diagnostic chart	X								
	Clinical examination	X								
	Preoperative conventional periapical X-ray	X								
	Preoperative photograph	X								
	Preoperative color assessment of affected tooth	X								
	Informed consent	X								
	Allocation			X						
	Impression for Radiographic stent	X								
Intervention	Access cavity		X							
	Minimal mechanical debridement		X							
	Irrigation		X							
	Antimicrobial dressing		X							
	Removal of antimicrobial dressing			x	X					
	Inducing bleeding			X	X					
	Placement of collagen plug			X	X					
	Placement of coronal plug material^®^			X	X					
	Access cavity sealing			X	X					
	Composite buildup									X
Assessment	Color assessment (baseline) then follow ups			X	X	x	x	X	X	x
	Clinical assessment			X	X	X	X	X	X	x
	Immediate post operative Digital Radiograph and follow ups			X	X				X	x
- Composite build-up (3 M, Filtek, Z350, United Kingdom) was done to all fractured teeth after the end of 12 month follow-up period to eliminate the masking effect of restoration on evaluation of discoloration.

In the follow-up visit the treated teeth were evaluated for:

Clinical parameters:

- Pain on biting: reported by asking the patient about the presence of pain while biting (Yes/No).
- Pain on percussion: detected by tapping the tooth with the back of an autoclavable mirror.
- Presence of swelling, sinus, or fistula: checked by visual examination and palpation of the labial vestibule and the palatal area related to every affected tooth.
- Mobility: was examined using the back of 2 autoclavable mirrors.

The unit of measuring these parameters was binary (present/absent).

Discoloration:

- Parental reporting of discoloration: by verbally asking parents whether they noticed visually the presence/absence of change in tooth color.
- Visual assessment of discoloration by two assessors (the main investigator and another clinical assessor) reported the presence/absence of tooth color change (Binary outcome) detected by visual examination.

- Cohen’s kappa value was used to assess the agreement between the 2 assessors.

c. Quantitative assessment of color change:

The spectrophotometer measured the color of teeth based on the Commission Internationale de I’Eclairage’s CIELAB color space system. The L* a* b* system allows color specification within a three-dimensional space where:

- The L* axis represents the degree of lightness within a tooth and ranges from 0 (black) to 100 (white).
- The a* plane represents the degree of green–red within a tooth, a* values ranges from red (+80a*) to green (−80a*).
- The b* plane represents the degree of blue–yellow within the tooth and b* values range from yellow (+80b*) to blue (−80b*).

- Three measurements were recorded for each follow-up then the mean color was calculated.
- The change in tooth color was calculated by monitoring changes in ΔL, Δ a, Δ b by subtracting the baseline measurements from the follow-up measurements.
- Delta E (ΔE) is the total color difference or the distance between two colors, (ΔE) was calculated according to the following equation:
  ΔE = ([L_x__ L_0_]^2^ + [a_x__a_0_]^2^ + [b_x__b_0_]^2^)^½^ [13, 14].
- The proposed limit for color difference adopted in this study was set at 3.7 Δ Ε units (perceptibility threshold) which means how much color change is considered perceptible, differences beyond this limit were considered clinically perceptible [14, 15].

Radiographic Parameters:

- Presence of external or internal root resorption.
- Assessment of periapical area: presence or absence of radiographic signs of infection.
- Root lengthening:

Root length was measured on the Digora software and the increase in root length and percentage of change in root length were calculated using equations according to Aly et al. [2].

*Increase in length* = *12* *m follow-up root length—immediate post-operative root length*

*Percentage of change in length* = *12* *m follow-up root length—immediate post-operative root length /immediate post-operative root length x 100.*

### Statistical analysis

The results of the present study were interpreted using per-protocol analysis.

Categorical data presented as frequencies and percentages were analyzed using the Chi-square test. Quantitative data were explored for normality using Kolmogorov-Smirnov and Shapiro-Wilk tests and were found to follow the normal distribution, so they were presented as mean and standard deviation (SD) and were analyzed using the independent t-test with the significance level set at *p* ≤ 0.05 for all tests. Cohen’s kappa value was used to assess the agreement between raters.

## Results

**Fig. 3:**
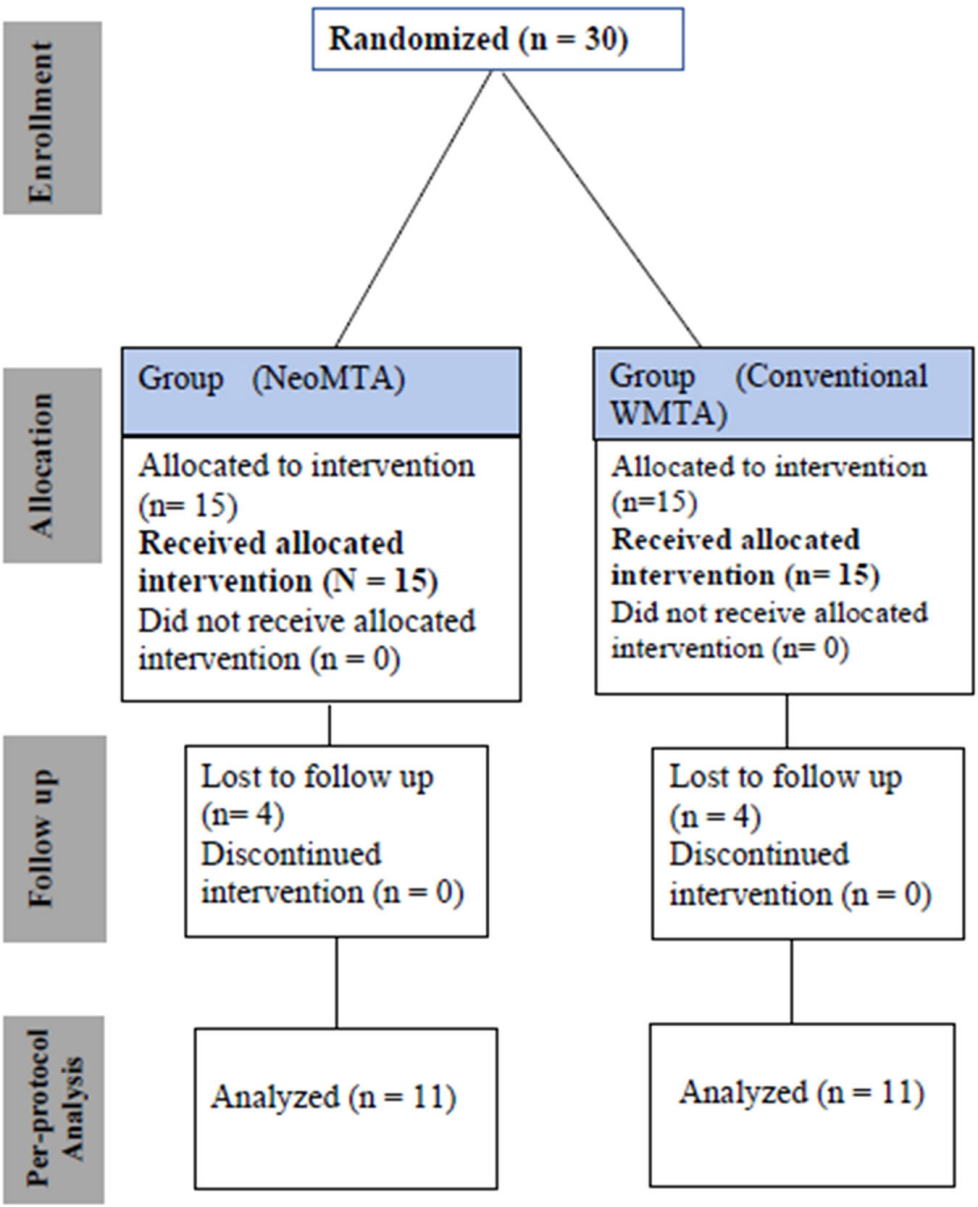
Flow Chart of the study population through the study.

During the follow-up period, four cases (26.7%) dropped out from each group and were excluded from the data analysis. Twenty children with 22 teeth completed the 12 months study period. The flow of the patients throughout the study is presented in Fig. 3.

Regarding the demographic data age and gender distribution among study subjects, were presented in Table 2 with no statistically significant difference within both groups (***p***-value= 0.443 and 0.771 respectively).

**Table 2:** Age and gender distribution among study between two groups.

Groups	Mean age	Gender %	
		Male	Female
Group (N)	9.07 ± 0.96	66.7%	33.3%
Group (W)	9.43 ± 1.55	53.3%	46.7%

Regarding the evaluation of clinical outcomes, all teeth showed normal clinical findings, there was a complete absence of signs and symptoms such as pain on biting, pain on percussion, swelling, sinus/fistula, and mobility in both groups during all follow-ups so both groups were (100%) clinically successful with no statistically significant difference between groups.

Concerning evaluation of discoloration, none of the parents reported discoloration at the 1-week follow-up, at 1 month only one parent in Group (N) reported the presence of discoloration in one tooth (9.1%) and two parents in Group (W) reported discoloration in 2 teeth (18.2%), at 3 and 12 months follow up only one parent in Group (N) reported discoloration at one tooth (9.1%) and three parents in Group (W) reported discoloration in 3 teeth (27.3%) and the difference between groups was not statistically significant (*p* value = 0.269).

The discoloration was assessed visually by two clinicians and there was an excellent agreement between both ratings of the clinical data (k = 1, *p* < 0.001). At 1 week no discoloration was detected in Group (N), while one tooth (9.1%) showed discoloration in Group (W). At 1 month, discoloration was detected in one tooth (9.1%) in Group (N) and three teeth (27.3%) in Group (W). At 3, 12 months follow-ups discoloration was detected in a single case (9.1%) in Group (N) and three cases (27.3%) in Group (W), and the difference between groups was not statistically significant (*p* value = 0.269).

The change in color between baseline and 12 months was quantified for each tooth by measuring the CIE L*a*b* values and calculation of ΔE.

At the end of 12 months, it was found that the mean ΔL value of Group (W) was more than Group (N), in a direction indicating decreased luminosity with no significant difference between groups.

The mean Δa value at the end of 12 months showed that Group (N) remained in the red values direction, while Group (W) showed a reduction in redness thus an increasing change towards the green direction. The alterations observed in the WMTA group were significantly greater compared with the other group.

The mean Δb value of Group (W) was more than Group (N), in a direction indicating a reduction of yellow color thus an increasing change towards the blue direction with no significant difference between groups. Mean and standard deviation values for ΔL, Δa, Δb are presented in Table 3.

**Table 3:** Mean and Standard deviation (SD) values for ΔL, Δa, Δb in both groups.

Parameter	Interval	(Mean ± SD)		*p*-value
		Group (N)	Group (W)	
**ΔL**	1 week- Baseline	−0.50 ± 1.65	−1.29 ± 2.29	0.365 ns
	1 month- Baseline	−0.69 ± 1.78	−2.04 ± 2.24	0.134 ns
	3 months- Baseline	−1.05 ± 2.80	−2.20 ± 2.45	0.317 ns
	12 months-Baseline	−1.01 ± 3.17	−2.80 ± 2.77	0.173 ns
**Δa**	1 week- Baseline	0.07 ± 0.34	−0.28 ± 0.48	0.070 ns
	1 month- Baseline	0.12 ± 0.40	−0.27 ± 0.42	0.037*
	3 months- Baseline	0.18 ± 0.28	−0.28 ± 0.44	0.011*
	12 months- Baseline	0.20 ± 0.40	−0.23 ± 0.52	0.041*
**Δb**	1 week- Baseline	−0.20 ± 1.10	−0.69 ± 1.05	0.294 ns
	1 month- Baseline	−0.56 ± 1.55	−0.69 ± 1.07	0.813 ns
	3 months- Baseline	−0.45 ± 1.71	−0.71 ± 1.36	0.693 ns
	12 months- Baseline	−0.40 ± 2.20	−0.71 ± 1.67	0.713 ns

*significant (*p* ≤ 0.05) ns; non-significant (*p* > 0.05).

**Fig. 4:**
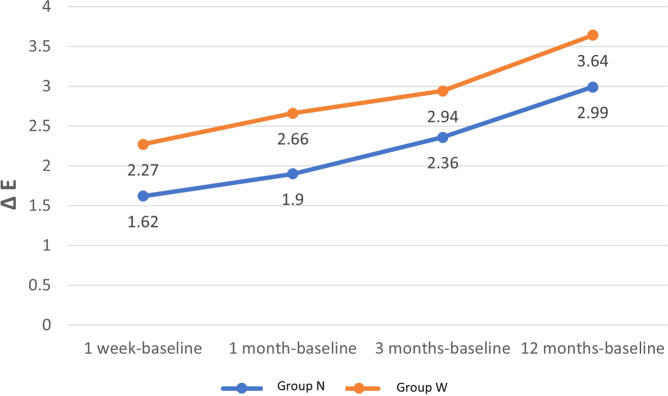
Line chart showing mean values for ΔE in both groups.

**Fig. 5:**
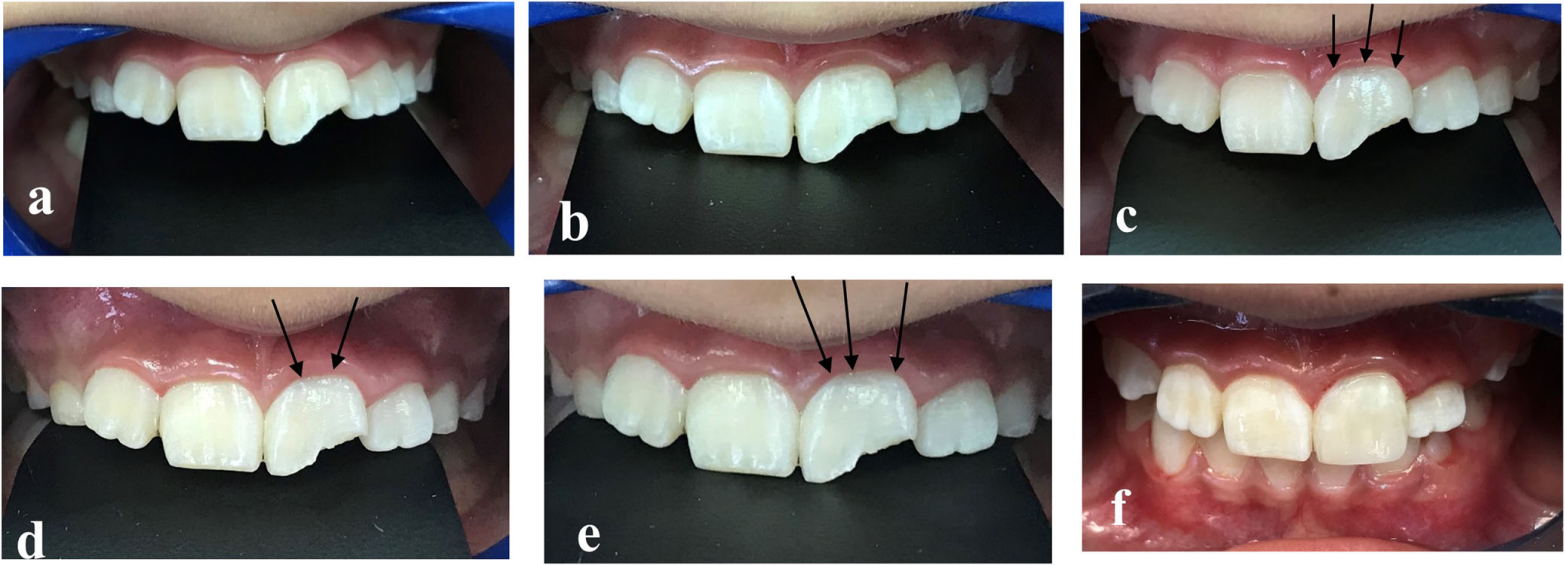
Clinical photographs of fractured upper left central incisor which was the single case that showed clinical discoloration in Group (N), change in color started to show at the 1 month follow up. **a** Baseline clinical photograph of upper left central incisor at second appointment, **b** Clinical photograph at 1 week follow up without discoloration, **c** Clinical photograph at 1 month follow-up showing shadow of cervical crown discoloration, **d** Clinical photograph at 3 months follow-up showing cervical crown discoloration, **e** Clinical photograph at 12 months follow-up showing cervical crown discoloration. **f** Clinical photograph showing composite buildup.

**Fig. 6:**
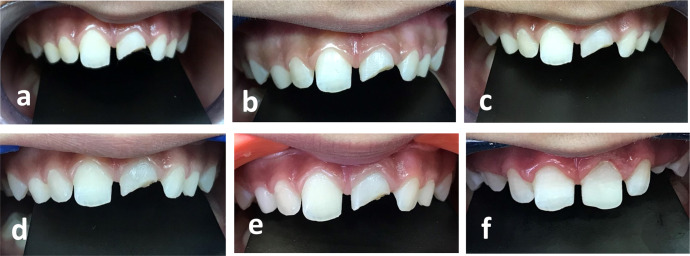
Clinical photographs of 9 years old child presented with traumatized upper left central incisor enrolled in Group (W) showing clinical change in color that started to show at the 1 month follow up. **a** Baseline clinical photograph at second appointment, **b** Clinical photograph at 1 week follow up without discoloration, **c** Clinical photograph at 1 month follow-up showing shadow of cervical crown discoloration, **d** Clinical photograph at 3 months follow-up showing cervical crown discoloration, **e** Clinical photograph at 12 months follow-up showing cervical crown discoloration. **f** Clinical photograph showing composite buildup.

The mean ΔE value at 12 months in the WMTA group was (ΔE = 3.64 ± 2.31) and NeoMTA group was (ΔE = 2.99 ± 2.56). No statistically significant difference between both materials during the 12 months (*p* value = 0.535). Mean values for ΔE are presented in Fig. 4. Clinical photographs showing the evaluation of discoloration in Group (N) and (W), Figs. 5 and 6.

The mean and standard deviation values for initial root length in mm were 11.694 (± 1.644) mm for Group (N) and 12.654(± 1.449) mm in Group (W). There was no significant difference in mean initial root length in both groups (*p*-value = 0.162).

**Fig. 7:**
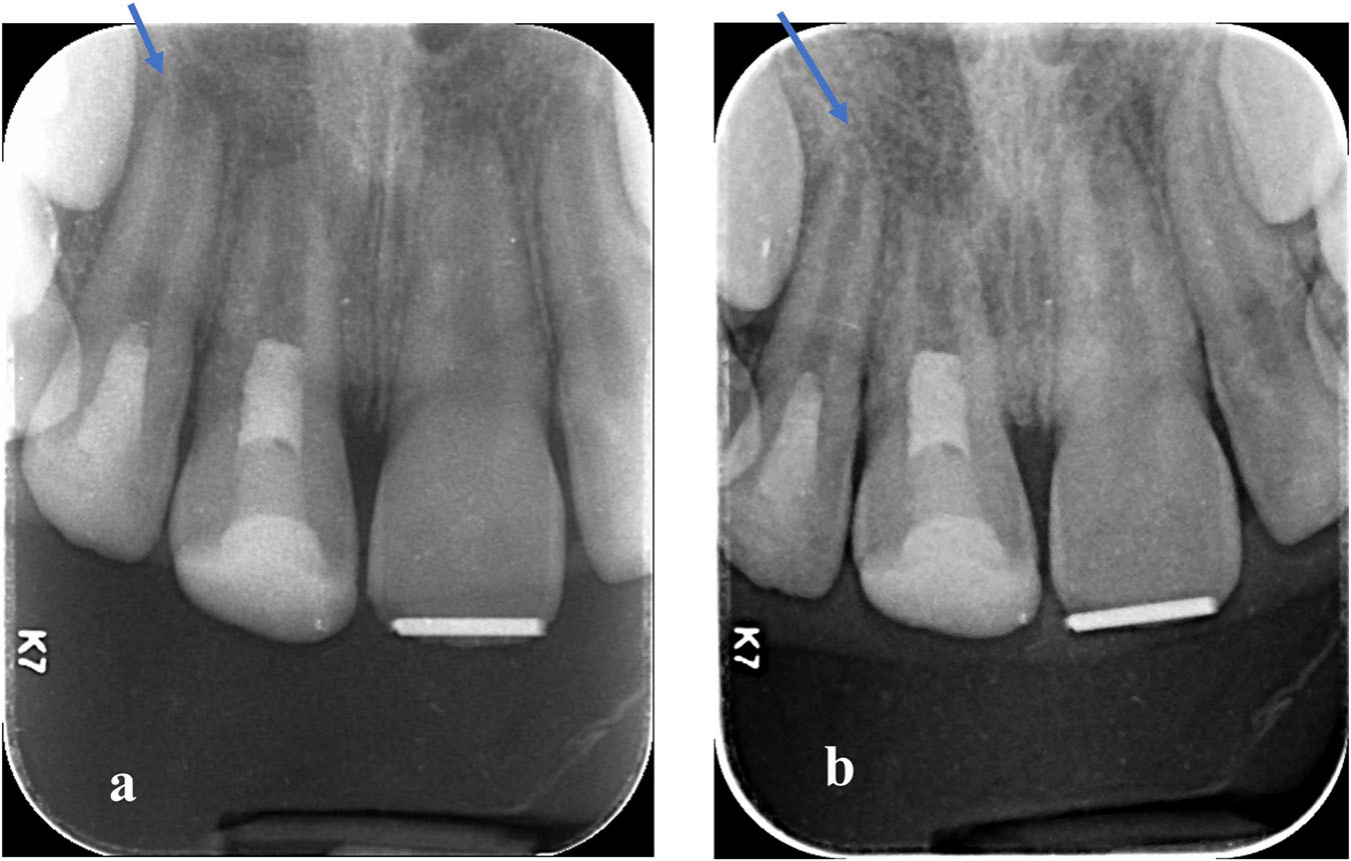
Digital periapical radiographic images of immature upper right lateral incisor representative for Group (N) showing continuing root lengthening. **a** Baseline immediate post-operative radiograph showing the immature root of upper right lateral incisor. **b** 12 months follow-up radiograph showing the increase in root length in upper right lateral incisor.

**Fig. 8:**
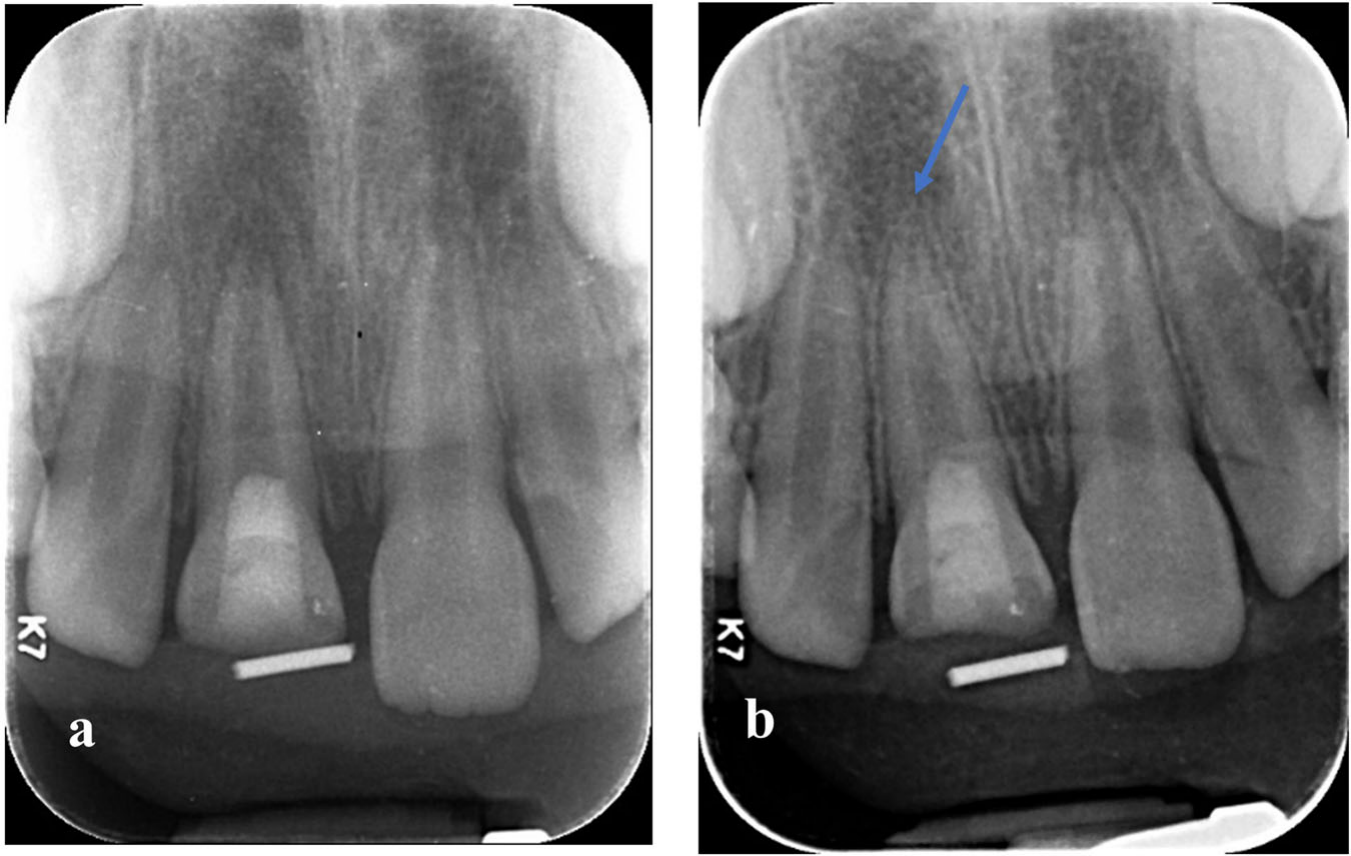
Digital periapical radiographic images of immature upper right central incisor representative for Group (W) showing continuing root lengthening. **a** Baseline immediate post-operative radiograph showing the immature root of upper right central incisor. **b** 12 months follow-up radiograph showing the increase in root length in upper right central incisor.

Continued root lengthening was observed in this study, the mean increase in root length in mm and percentage between 12 months follow-up and pre-operative root length in Group (N) was 1.03 (± 0.97) mm, 8.52(± 3.33)% and in Group (W) was 1.04 (± 0.86) mm, 8.64(± 4.30)% with no significant difference between both groups. Mean and Standard deviation (SD) values are shown in Table 4. Digital radiographs showing an increase in root length in Group (N) and (W), Figs. 7, 8.

**Table 4:** Mean and Standard deviation (SD) values for change in root length in (mm) and (%) in both groups.

Parameter	(Mean ± SD)		*p* value
	Group (N)	Group (W)	
Difference (mm)	1.03 ± 0.97	1.04 ± 0.86	0.979 ns
Percentage change (%)	8.52 ± 3.33	8.64 ± 4.30	0.973 ns

Regarding the radiographic evaluation, all teeth in both Groups (N) and (W) were free of internal and external root resorption also there was a complete absence of any radiographic signs of infection, accordingly the overall clinical and radiographic success rate was 100% for both Groups (N) and (W).

## Discussion

Revascularization of immature permanent teeth has become an important part of endodontic treatment modalities. Despite its high success rate clinically and radiographically, the concerns about post-revascularization tooth discoloration were increasing [7]. This outcome is unfavorable and significantly impacts the quality of life in children negatively, therefore materials with the lowest possible staining potential should be considered in the esthetic zone.

The cause of post-revascularization discoloration is still debatable, but most fingers point to the presence of bismuth oxide in the conventional WMTA and its reaction with dental tissues, blood products, and irrigants used in revascularization procedures [16,17]. Although WMTA was developed to overcome tooth discoloration encountered with the use of the Grey mineral trioxide aggregate (GMTA), several in vitro studies had reported evidence of tooth discoloration [14, 18].

Accordingly, NeoMTA was suggested as coronal plug material in the intervention group, since it is a pure WMTA that is marketed by having a new non-staining formulation, due to replacing the radiopacifier (Bi_2_O_3_) with (Ta_2_O_5_), moreover, it has been reported that it has better handling characteristics and forms putty consistency upon mixing [8]. Therefore, it was recommended to investigate the material performance in various clinical applications.

The inclusion and exclusion criteria of the present study have been determined according to the AAE [12] clinical considerations for regenerative procedures. Teeth showing severe coronal fracture were excluded from the study because the pulp space won’t be available for post retention and the VITA Easyshade probe’s diameter is 5 mm so, at least 5 mm of intact cervical enamel was needed to evaluate color changes [19]. Teeth with luxation injuries were excluded since the stem cells from the apical papilla (SCAPs) and Hertwig epithelial root sheath (HERS) might be damaged [20].

The exclusion of cases with unacceptable discoloration compared with the contralateral tooth was done in order not to affect the evaluation of discoloration that might occur. The unacceptable discoloration limit for exclusion of cases (ΔE more than 5) was determined by following the intraoral perceptibility and acceptability tolerances for color mismatch reported in the literature, a difference of ΔE starting from 5.5 to 6.8 was required for dentists to find the color mismatch sufficiently unacceptable to suggest further treatment to improve it [21, 22].

The radiographic standardization was done by the construction of a radiographic stent for bite registration to facilitate consistent positioning of the film. Moreover, (XCP) alignment system was used for a paralleling technique to allow comparisons of the digital radiographs [2, 23]. A radio-opaque marker of known dimension was embedded in the acrylic resin stent to help in the determination and compensation of the magnification factor of the radiographic images thus minimizing errors in the measurement of root length [11].

Treatment procedures of the selected teeth were performed according to AAE [12] clinical considerations for a regenerative procedure. Lower concentrations of NaOCl (1.5 % concentration) were advised and recommended by **AAE** [12] to keep the balance between sufficient disinfection and preservation of SCAPs. Since it has been proven that using higher concentrations of NaOCl has a profound negative effect on the survival and differentiation of SCAPs or expression of proteins of the dentin matrix that are involved in promoting tissue regeneration [24–26].

It was recommended by the **AAE** [12] to set the second appointment within 1–4 weeks from the first appointment according to the assessment of the response to initial treatment, Since it was a must to standardize the time of the second appointment for all patients to limit any variables the maximum time interval given by the AAE was set for all patients to be able to take a decision either to proceed for a second visit or consider additional treatment time with antimicrobial.

The baseline color assessment was done at the beginning of the second appointment before the teeth are exposed to dehydration as recommended by Burki et al. [27] since most dental procedures lead to teeth dehydration which alters tooth shade and shows perceivable color change causing errors in color assessment.

Clinical follow-up protocol was planned to be after 1 week, 1 month, 3 months, 6 months, and 12 months in accordance with previous literature assessing and evaluating discoloration quantitatively [10, 28, 29].

The covid-19 pandemic lockdown caused unique challenges for the clinical trial community around the world [30]. The effect of lockdown was noticed in the present study in the 6 months’ records as all patients were not able to attend the clinic. Also, it led to the drop out of 5 children having 8 treated teeth as their parents were unwilling to come for completion of the follow-up period even after the end of lockdown although they didn’t have their final composite build-up. Since the dropout was within the number calculated in the sample size, there was no need to compensate any of the dropout patients.

In the present study, boys to girls distribution were 18 boys (60%) and 12 girls (40%) respectively. This finding could be related to the results observed by El-Kenany et al. [31] that indicated a gender difference in injury rates among 8–12 years old school children in Egypt. This might be related to boys’ tendency of choosing more energetic, active, and vigorous outdoor games.

Regarding the clinical outcomes, both groups were (100%) clinically successful as there were a complete absence of pain on biting, pain on percussion, swelling, sinus/fistula, and mobility in both groups during all follow-ups. This could be explained by the standardized disinfection protocol and the good coronal seal used in both groups. The clinical outcomes were consistence with the findings of Aly et al. [2] and Rizk [23]. On the contrary, a study done by Linsuwanont et al. [32] reported a lower clinical success rate as (76%) of cases were successful, the author explained that the root canal disinfection protocol was not effective.

Evaluation of discoloration was done in the present study by 3 methods. Concerning the discoloration as reported by the parents, only one parent (9.1%) in Group (N) and surprisingly only three parents (27.3%) in Group (W) reported discoloration at the end of 12 months. This result was not in line with Nazzal et al. [33] who reported that 7 parents out of 12 observed discoloration although double antibiotic paste (DAP) and non-bismuth-containing Portland cement were used.

Visual assessment of discoloration by the two assessors at 12 months was similar to the results reported by parents. However, assessors reported discoloration at an earlier time this was due to the fact reported by Vichi et al. [34] that skilled operators perceive color difference at earlier levels than untrained observers.

Although NeoMTA showed less discoloration potential compared to WMTA angelus, there was no statistically significant difference between both groups. Little information is currently available on NeoMTA, the results of the NeoMTA group were in line with the conclusion of the invitro study done by Camilleri, [35] that NeoMTA has better color stability than conventional WMTA because of its new formulation that replaced the bismuth oxide with tantalum oxide. The result of discoloration outcome of the WMTA in the present study was in agreement with Nagata et al. [36] that reported only 3 teeth out of 11 (27.3%) showed discoloration in revascularization protocol using non-staining intracanal medication and WMTA (Angelus) that was placed above collagen matrix. On the contrary, other clinical studies reported a higher percentage of discoloration in revascularization procedures using non-staining intracanal medication and WMTA as Kahler et al. [37] reported discoloration in 10 cases out of 16 and Aly et al. [2] reported discoloration in (53.84%) of cases.

These contradictory results might be explained by slightly different clinical procedures. First, it was found that in the present study WMTA was not in direct contact with the blood clot, it was placed on collagen matrix, while in the aforementioned clinical studies, WMTA was in direct contact with blood without collagen matrix. The use of synthetic resorbable matrix over the blood clot, separating it from the barrier materials was suggested by Žižka et al. [38] and Wei X et al. [26] as a way for minimizing discoloration in REP, but no available clinical trials confirmed this postulation.

The later postulation was supported by Lenherr et al. [10] and Žižka et al. [38] that WMTA and all calcium silicate-based cements containing bismuth oxide or other radiopacifiers showed exacerbated discoloration after contact with blood due to absorption of blood disintegration products into the porosities of freshly unset materials, Thus, concluded that allowing the coronal plug materials to set away from blood is beneficial in decreasing discoloration.

The second explanation for the conflicting results of WMTA might be related to the position of WMTA in relation to CEJ. In the present study, WMTA was placed accurately just below CEJ with the aid of collagen matrix, While Kahler et al. [37] and Aly et al. [2] reported that controlling the position of WMTA above the blood clot was technically difficult and the authors justified that one of the possible causes of discoloration was the presence of WMTA above the CEJ. This was in line with Žižka et al. [38] recommendation that placing the coronal barrier just below CEJ makes the discoloration of tooth structure be covered by bone or gingiva.

Concerning the quantitative assessment of color change. The smaller the ΔE value, the lower the color difference between the initial and final color of the tooth over time. The mean ΔE value showed no statistically significant difference between both materials during the 12 months, this might be explained by that ΔE quantifies the color change but not the direction. It is only when the different components of color, L* a * b*, are analyzed individually that the nature of the color change can be identified.

The direction of color change in the WMTA group towards decreased luminosity, and reduction of redness and yellowness thus increasing the tendency towards green and blue direction agreed with Ioannidis et al. [14] and Esmaeili et al. [39], however, the present study showed less values of change. This difference in values could be explained by different study settings, as those studies were performed in laboratory conditions, which do not reflect the actual clinical conditions. As in the present study WMTA was applied carefully in areas of aesthetic concern just below CEJ to reduce the risk of material-induced tooth discoloration and the discoloration of tooth structure might be covered by bone or gingiva.

Esmaeili et al. [39] clarified that variable amounts of color change were reported with the same formulation of WMTA would be as a result of different thicknesses of the remaining tooth structure, colorimetric method of measurement, and material application methods. Accordingly, direct comparisons and interpretation of the available data concerning ΔE and different components of color, L* a * b* values were difficult to be performed due to the different experimental methodologies.

The proposed limit for color difference adopted in this study was set at 3.7 ΔE units (perceptibility threshold), as there was a lack of consensus in the literature about the perceptibility threshold set value ranging between 2.6 and 3.7, it was set at the highest value in the present study since it was suggested that clinicians are more tolerant of color difference in a clinical scenario than under controlled in vitro conditions [15]. Therefore, although the mean ΔE value was more in the WMTA group than the NeoMTA group both materials were below the perceptibility threshold.

In the present study, the ΔE value of WMTA was in agreement with the invitro study conducted by Rouhani et al. [40] and Beatty & Svec, [41] that reported the ΔE value of WMTA was 3.54, and 2.6 respectively. While in terms of visual perception, they were not consistence together since the perceptibility threshold value was different.

On the contrary Ioannidis et al. [14] reported a higher ΔE value for WMTA than the present study this might be explained by Žižka et al. [38] that if recommendations are meticulously followed the level of discoloration of teeth can be minimized below the human eye threshold.

On the other hand, a clinical study done by Nazzal et al. [33] reported that post-revascularization discoloration occurred with ΔE value = 7.9, although the intracanal medication was minocycline free and the coronal plug material was non-bismuth containing Portland cement that was placed directly above blood clot. This supports the postulation that the cause of discoloration is multifactorial and doesn’t depend solely on the radio pacifier of coronal plug material or the intra-canal medication.

Regarding the radiographic outcomes, the mean initial pre-operative root length of teeth showed no statistically significant difference between both groups, which indicates that the 2 groups were comparable and there was no variability between the selected teeth in both groups.

The change in root length results were generated in millimeter units and as percentage change from preoperative values as well, this was to provide a more conservative analysis as each case is normalized to its pre-operative measurement. In addition, the units of percentage change provide a clinically meaningful outcome when considering the impact of REPs [2].

Continued root lengthening observed in this study was in consistence with the results of Nagy et al. 2014 [42] reported the mean increase in root length was 0.8 ± (0.5) mm, 5% and also was comparable to Aly et al. [2] results as the mean increase in root length was 0.7 (± 0.23) mm, 5.02% in immature teeth treated with the same protocol as the present study using (DAP) and WMTA.

On the contrary Mittmann et al. [43] reported that the percentage of increase in root length was 0.96% during 22 months follow-up. This contradictory data can be explained by different case selections as the aforementioned study done by Mittmann et al. [43] included luxation injuries and replanted avulsed teeth that have a higher risk of (HERS) or apical papilla injury, which are described as the most important elements controlling continuing of root development.

There was no statistically significant difference in the increase in root length in mm and percentage between the two groups. This might be explained by the postulation of Kharchi et al. [25] that regardless of the REP details, the single most important variable for further root development in the regeneration procedure is the disinfected root canal, whenever a sufficiently disinfected environment was created this allows a biological response of appropriate cells to support the continued root development.

All teeth in both groups were free of internal and external root resorption. However, Mittmann et al. [43] reported (56.3%) of teeth developed root resorption, but this could be explained by different case selections as resorption is more likely to be due to great damage to periodontal ligament due to luxation injuries rather than by the revascularization treatment.

All teeth in both groups showed a complete absence of any radiographic signs of infection, accordingly, all the teeth in both groups were having 100% radiographic success and 100% overall success rate, this was in agreement with Kahler et al. [37] and Rizk, [23] reported 100% overall clinical and radiographic success rate, and was comparable to systematic review and meta-analysis done by Koç & Del Fabbro, [20] reported that overall success rate of revascularization procedures in teeth with traumatic injuries was 94.8%. This shows that the primary outcome of revascularization (elimination of clinical symptoms and resolution of apical periodontitis) can be easily achieved as long as the infection is controlled [4].

There was no statistically significant difference in the overall success rate between the two groups but this could be explained by the fact reported by Khan et al. [8] that NeoMTA is a pure MTA with a new formulation having alternative radiopacifiers, so it is exhibiting physical and chemical properties compared with conventional WMTA.

**Study limitations**

1. COVID-19 pandemic lockdown affected the attendance of patients to follow-up.
2. Comparing and interpreting the results of the present study with other clinical studies was difficult because clinical protocols among the previous studies were widely varied.
3. The data from in-vitro or in-vivo studies on NeoMTA was scarce.

**What this paper adds**

1. Both NeoMTA and conventional WMTA were successful coronal plug materials in the revascularization of non-vital immature permanent teeth achieving a high level of clinical and radiographic success.
2. NeoMTA is a promising coronal plug material that can be used for revascularization procedures in the esthetic zone as it showed less discoloration potential compared with conventional WMTA, however, there was no statistically significant difference between both groups.
3. Coronal discoloration in the regenerative endodontic procedure is a multifactorial process not only related to the composition of materials used but other factors might minimize or exacerbate the discoloration potential.
4. If clinical guidelines and recommendations were scrupulously followed, the degree of teeth discoloration might be reduced below the human eye perceptibility threshold.

## Data Availability

Data are available upon reasonable request.
